# Stress in obstructive sleep apnea

**DOI:** 10.1038/s41598-021-91996-5

**Published:** 2021-06-16

**Authors:** Jasmine L. Wong, Fernando Martinez, Andrea P. Aguila, Amrita Pal, Ravi S. Aysola, Luke A. Henderson, Paul M. Macey

**Affiliations:** 1 UCLA School of Nursing, University of California at Los Angeles, 700 Tiverton Avenue, Los Angeles, CA 90095 USA; 2 Department of Medicine (Division of Pulmonary and Critical Care), David Geffen School of Medicine at UCLA, University of California at Los Angeles, Los Angeles, CA 90095 USA; 3 Department of Anatomy and Histology, Sydney Medical School, University of Sydney, Sydney, Australia; 4 Brain Research Institute, David Geffen School of Medicine at UCLA, University of California at Los Angeles, Los Angeles, CA 90095 USA

**Keywords:** Sleep disorders, Psychiatric disorders

## Abstract

People with obstructive sleep apnea (OSA) often have psychological symptoms including depression and anxiety, which are commonly treated with anti-depression or anti-anxiety interventions. Psychological stress is a related symptom with different intervention targets that may also improve mental state, but this symptom is not well characterized in OSA. We therefore aimed to describe stress in relation to other psychological symptoms. We performed a prospective cross-sectional study of 103 people, 44 untreated OSA (mean ± s.d. age: 51.2 ± 13.9 years, female/male 13/31) and 57 healthy control participants (age: 46.3 ± 13.8 years, female/male 34/23). We measured stress (Perceived Stress Scale; PSS), excessive daytime sleepiness (Epworth Sleepiness Scale; ESS), depressive symptoms (Patient Health Questionnaire; PHQ-9), and anxiety symptoms (General Anxiety Disorder; GAD-7). We compared group means with independent samples t-tests and calculated correlations between variables. Mean symptom levels were higher in OSA than control, including PSS (mean ± s.d.: OSA = 15.3 ± 6.9, control = 11.4 ± 5.5; *P* = 0.002), GAD-7 (OSA = 4.8 ± 5.0, control = 2.1 ± 3.9; *P* = 0.02), PHQ-9 (OSA = 6.9 ± 6.1, control = 2.6 ± 3.8; *P* = 0.003) and ESS (OSA = 8.1 ± 5.3, control = 5.0 ± 3.3; *P* = 0.03). Similar OSA-vs-control differences appeared in males, but females only showed significant differences in PHQ-9 and ESS, not PSS or GAD-7. PSS correlated strongly with GAD-7 and PHQ-9 across groups (R = 0.62–0.89), and moderately with ESS. Perceived stress is high in OSA, and closely related to anxiety and depressive symptoms. The findings support testing stress reduction in OSA.

## Introduction

People with obstructive sleep apnea (OSA) often report psychological symptoms beyond the characteristic excessive daytime sleepiness, including depression and anxiety^1–7^. Such accompanying symptoms are frequently the initial reason people visit the healthcare professional who subsequently refers them for a sleep study that results in a diagnosis of OSA^2^. Once a person is diagnosed with OSA, psychological symptoms are usually addressed either indirectly by treating the OSA with continuous positive airway pressure (CPAP) therapy or directly through depression and anxiety interventions^8^. However, CPAP typically improves but does not normalize depression or anxiety^9–11^, and the standard treatments for depression or anxiety may be unwarranted given that depressive and anxiety symptoms in OSA usually do not rise to the level of a diagnosable mental health disorder. Furthermore, some antidepressant and anti-anxiety medications worsen risk factors for OSA^12,13^.

An alternative approach to addressing psychological symptoms in OSA may be through stress-reduction interventions. Anxiety and depression are related to psychological stress, with measures of increased psychological stress often including parameters associated with increased anxiety and depression symptoms^14^. Whilst treatment approaches targeting stress differ from those targeting depressive or anxiety symptoms^15,16^, given the overlap, interventions that reduce psychological stress may be effective in also addressing OSA related anxiety and depression symptoms.

Stress here is defined as a state of mental or emotional strain or tension. Such stress is linked with poor health outcomes^17^, with two thirds of the United States population indicating their health is affected by stress^18^. Stress in people who already have an existing chronic health condition predicts poorer outcomes, suggesting that addressing stress in OSA could lead to improved outcomes^19^. There is also evidence of sex-specific consequences of stress, which are relevant given the different prevalence and characteristics of OSA in men and women^20^. One mechanism linking OSA and stress could be injury in brain regions known to be altered in individuals with chronic psychological stress^21,22^.

There is currently little known about the prevalence of psychological stress in OSA and, as a consequence, stress relief regimens are not commonly considered for individuals with OSA. The motivation for this study was therefore to evaluate the incidence of psychological stress, and associations with depression and anxiety symptoms in OSA. Our objective was to determine in a cross-sectional sample if psychological stress is higher in patients with OSA compared with healthy participants, and describe relationships of stress with sleepiness, depression, and anxiety, with sex-specific consideration. We also aimed to compare those relationships between people with and without OSA. We hypothesized that 1) OSA is associated with higher psychological stress compared with controls; 2) stress is correlated with other psychological symptoms; and 3) the mean stress levels and magnitudes of those correlations differ between men and women.

## Methods

We performed a prospective cross-sectional study on people with untreated OSA and healthy participants from 2017 to 2020. We used self-report surveys to evaluate psychological symptoms, and screening and home sleep studies to assess sleep apnea status. The procedures were approved by the UCLA Institutional Review Board (IRB #10–001012 and #18–000184), and all participants provided written informed consent. All methods were carried out in accordance with relevant guidelines and regulations.

Participants were recruited via paper fliers placed at the UCLA Sleep Disorders Center, the UCLA campus and surrounding community, and digital fliers on UCLA Health Sciences sites advertising research studies. We studied 44 individuals with untreated OSA and 57 healthy, control participants without major comorbidities (details in Table 1). Participants with OSA were recruited in conjunction with the UCLA Sleep Disorders Center, and diagnosed according to the 2012 American Academy of Sleep Medicine criteria^23^. Two-night home sleep apnea testing (HSAT) with an ARES device^24^ was used to calculate the respiratory event index (REI) using similar criteria as the apnea–hypopnea index (AHI) and OSA diagnosed based on the 2012 American Academy of Sleep Medicine (AASM) scoring guidelines^23^. The ARES has FP1 and FP2 for deriving EEG, EOG and EMG but does not qualify for the AASM definition of HSAT sleep vs. wake. Thus, the REI is apnea/hypopneas per hour of recording time, not sleep time. The ARES device captures airflow using a nasal cannula and pressure transducer, and apnea is cessation (> 90% reduction) in flow for ≥ 10 s, and hypopnea ≥ 50% reduction in flow for ≥ 10 s. The criteria are for REI apneas and hypopneas are a minimum 4% desaturation. The scoring assigned to participants was based on the average over the single night with the longest valid recording time. A minimum of 4 h of successful data acquisition during sleep was required, with a technologist verifying the continuous recording of all signals and sleep state based on EEG. If these criteria were not met, participants were offered coaching on using the ARES device and invited to attempt a second 2-night study. Inclusion criteria for the patient group included a diagnosis of mild (REI 5–14), moderate (REI 15–29) or severe OSA (REI ≥ 30).

**Table 1:** Characteristics of participants.

Subject Information	All			Female			Male		
	Control	OSA	*P*	Control	OSA	*P*	Control	OSA	*P*
†	Mean ± s.d. [range]	Mean ± s.d. [range]		Mean ± s.d. [range]	Mean ± s.d. [range]		Mean ± s.d. [range]	Mean ± s.d. [range]	
N	57	44		34	13		23	31	
Sex	34♀ 23♂	13♀ 31♂	0.006	-	-	-	-	-	-
Age (years)	46.3 ± 13.8 [21.3–71.0]	51.2 ± 13.9 [25.6–77.7]	0.08	44.8 ± 13.8 [21.4–71.0]	60.1 ± 12.4 [40.3–77.7]	0.001	48.5 ± 13.7 [23.5–69.1]	5.0 ± 5.5 [0–21]	0.5
BMI (kg/m ^2^)	25.6 ± 4.9 [17.4–40.8]	32.1 ± 6.6 [22.3–47.0]	< 0.001	24.4 ± 4.5 [17.4–37.6]	31.3 ± 6.1 [23.9–43.1]	0.002	27.3 ± 5.1 [19.8–40.8]	32.5 ± 6.8 [22.3–47.0]	0.002
**Sleep**	N = 14/57			N = 11/34			N = 3/23		
REI (events/hour)	2.1 ± 2.3 [0–4]	24 ± 21 [6–107]	n/a	1.5 ± 1.2 [0–4]	18 ± 15 [7–53]	n/a	2.7 ± 1.5 [1–4]	27 ± 22 [6–107]	n/a
SaO_2_ Baseline (%)	96.3 ± 0.4 [95.5–97.0]	94.8 ± 1.6 [90.0–97.0]	n/a	96.3 ± 0.5 [95.5–97.0]	95.1 ± 1.1 [93.0–96.5]	n/a	96.3 ± 0.4 [96.0–97.0]	94.6 ± 1.8 [90.0–97.0]	n/a
SaO_2_ Nadir (%)	89.1 ± 2.8 [84.8–93.4]	83.8 ± 5.6 [72.0–92.0]	n/a	89.2 ± 3.0 [84.8–93.4]	84.8 ± 4.8 [75.1–92.0]	n/a	88.8 ± 2.2 [86.3–90.5]	83.4 ± 5.9 [72.0–92.0]	n/a
**Symptoms**									
Stress (PSS)	11.4 ± 5.5 [4–26]	15.3 ± 6.9 [4–27]	0.002	12.4 ± 5.3 [5–26]	15.2 ± 6.6 [6–26]	0.2	9.8 ± 5.5 [4–25]	15.3 ± 7.1 [4–27]	0.003
Anxiety (GAD-7)	2.1 ± 3.9 [0–21]	4.8 ± 5.0 [0–21]	0.02	2.35 ± 3.50 [0–14]	4.2 ± 3.6 [0–11]	0.1	1.6 ± 4.5 [0–21]	5.0 ± 5.5 [0–21]	0.02
Depression (PHQ-9)	2.6 ± 3.8 [0–19]	6.9 ± 6.1 [0–25]	0.003	2.68 ± 2.94 [0–13]	5.8 ± 4.6 [0–15]	0.04	2.6 ± 5.0 [0–19]	7.4 ± 6.6 [0–25]	0.004
Excessive daytime sleepiness (ESS)	5.0 ± 3.3 [0–14]	8.1 ± 5.3 [0–22]	0.02	5.0 ± 3.1 [0–11]	8.3 ± 4.0 [3–17]	0.02	5.0 ± 3.6 [0–14]	8.1 ± 5.8 [0–22]	0.02

Mean, standard deviation and range for continuous variables are shown for OSA and control groups separately for combined-sex (All), female and male. P-values for OSA-control comparisons are based on two-tailed independent samples t-test for continuous variables and Chi-square for sex. n/a: tests not performed due to subset of control participants with sleep study.

^†^ *BMI* body mass index, *ESS* Epworth Sleepiness Scale, *GAD-7* Generalized Anxiety Disorder scale, *PHQ-9* Patient Health Questionnaire, *PSS* Perceived Stress Scale, *REI* respiratory event index, SaO_2_ oxygen saturation. ♀ = female, ♂ = male.

Control participants underwent a three-step screening for sleep disordered breathing and other sleep disorders. During enrollment, a phone screening included questions about diagnosed sleep disorders, sleep complaints, or snoring. After enrollment, participants completed an online questionnaire that included questions about sleep disorders, sleep complaints, and daytime sleepiness. Participants reporting daytime sleepiness or other sleep complaints completed the HSAT through the UCLA Sleep Disorders Center to test for OSA. Most sleep screenings were performed weeks prior to enrollment and data collection (mean 83 days). When possible, OSA status was confirmed by reviewing the participants’ existing sleep study records, and some participants had been offered CPAP, but none had successfully initiated treatment, and no other apnea treatments (dental appliance, surgery) were used. Exclusion criteria for all participants included: current (< 1 year) tobacco use; current (< 3 months) use of psychotropic medications; major head injuries; mental illness other than unipolar depression or generalized anxiety. Subjects were recruited for study procedures that included brain imaging for a subset of participants, so exclusion criteria included MRI contraindications of no claustrophobia, no metal implants, and weight of less than 125 kg.

Eligible participants were scheduled for a visit where they completed the following questionnaires: (i) Perceived Stress Scale (PSS), a measure of the degree to which situations in an individual’s life are appraised as stressful, designed to assess how unpredictable, uncontrollable, and overloaded individuals find their lives. A PSS score of 0 to 13 indicates low stress, 14 to 26 moderate stress, and 27 to 40 high perceived stress^25^. (ii) Epworth Sleepiness Scale (ESS), a measure of excessive daytime sleepiness. An ESS score of 0 to 10 normal daytime sleepiness, 11 to 12 mild excessive daytime sleepiness, 13 to 15 moderate daytime sleepiness, and 16 to 24 severe excessive daytime sleepiness^21^. (iii) Patient Health Questionnaire nine-item (PHQ-9), a measure of depressive symptoms. A PHQ-9 score of 0 to 4 indicates minimal or no depressive symptoms, 5 to 9 is mild depression, 10 to 14 is moderate depression, 15 to 19 is moderately severe depression, and 20 to 27 indicates severe depression^26^. (iv) General Anxiety Disorder seven-item (GAD-7), a measure of anxiety symptoms. A GAD-7 score of 0 to 4 indicates no or minimal anxiety, 5 to 9 mild anxiety, 10 to 14 moderate anxiety, and 15 to 21 severe anxiety^27^. These surveys are widely used, freely available, and have moderate to strong reliability and validity in the general population, although the PSS, PHQ-9 and GAD-7 have not been assessed in OSA samples^25–30^. We also collected each participant’s age and sex, and calculated body mass index (BMI) based on height and weight recorded at the visit.

We reported the mean, standard deviation, and range. We used MATLAB’s (The Mathworks Inc., Natick, MA) “boxplot” function to visualize measures by group; this function displays median, lower and upper quartiles, range without outliers, and outliers defined as values either 1.5 times the interquartile range above the upper quartile or below the lower quartile (see MATLAB “boxplot” documentation). We compared group mean levels with independent samples t-tests, and performed Pearson’s correlations between stress and other variables. For hypothesis testing, we set the threshold at *P* = 0.05. For each measure, we performed independent sample t-tests to compare means between OSA and control groups for (1) all subjects, (2) females, and (3) males. To compare correlations between OSA and control, we used the Fisher r-to-z transformation. Since all variables other than PSS have already shown differences in OSA, we did not control for multiple comparisons since we had prior hypotheses that these would differ. We examined correlations in all groups between PSS and the other variables. In the OSA groups, we assessed correlations of PSS with REI and SaO_2_ nadir. In addition to calculating within-group correlation values, we tested for OSA and control differences in correlations.

## Results

Subject characteristics are shown in Table 1. We screened 235 people to obtain the final sample of 101; most excluded people did not meet criteria, and one was unable to complete the HSAT. Three participants performed a third night of HSAT to achieve 4 h of sufficient quality data during sleep. There was no significant difference in age between the combined control and OSA groups, although dividing by sex revealed significantly older female OSA compared with female control subjects. Additionally, sex distribution differed, with more females in the control group. BMI was higher in OSA.

**Figure 1:**
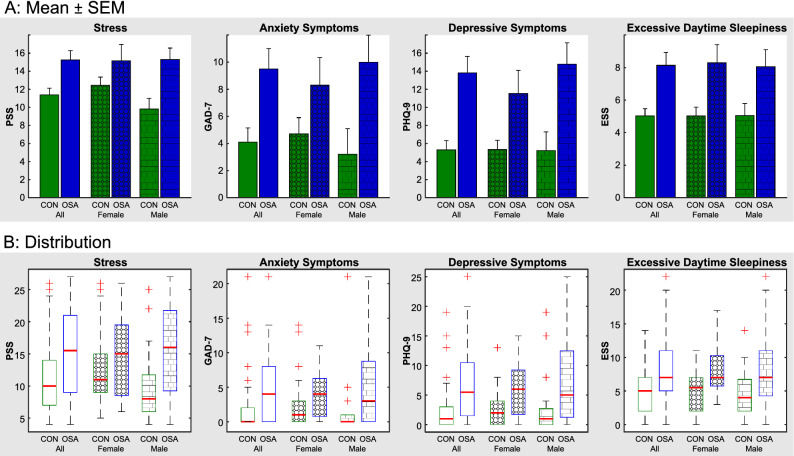
Group Means and distributions of symptoms in OSA. (**A**) Bar graphs of mean ± SEM values and (**B**) distributions of four psychological variables for each group. Green = control, blue = OSA. Plain fill = combined sexes, circle hatching = females, rectangular hatching = males. B: boxplots show lower and upper quartiles (box), median (red line in box), range without outliers (black dashed lines), and outliers (red crosses), where outliers are values more than 1.5 times the interquartile range above the upper quartile or below the below the lower quartile. PSS = Perceived Stress Scale; GAD-7 = Generalized Anxiety Disorder scale; PHQ-9 = Patient Health Questionnaire; ESS = Epworth Sleepiness Scale.

Mean levels of stress (PSS) were higher in the combined OSA group compared with controls (mean ± s.d.: OSA = 15.3 ± 6.9, control = 11.4 ± 5.5; *P* = 0.002). On average, OSA subjects had PSS values considered indicative of moderate stress (14–26) whereas controls subjects had values in the low stress (≤ 13) category. Although PSS values were not significantly different between OSA and control females, this lack of significance was driven by a higher level in female controls, since PSS values were almost identical in OSA females compared with OSA males (mean 15.2 versus 15.3, respectively). Levels of symptoms other than stress were all higher in OSA than control for the combined-sex groups (Table 1, Fig. 1), including anxiety (GAD-7; OSA = 4.8 ± 5.0, control = 2.1 ± 3.9; *P* = 0.02), depression (PHQ-9; OSA = 6.9 ± 6.1, control = 2.6 ± 3.8; *P* = 0.003) and excessive daytime sleepiness (ESS; OSA = 8.1 ± 5.3, control = 5.0 ± 3.3; *P* = 0.03). On average, OSA subjects had depressive symptom levels in the mild range whereas controls had depressive symptoms in the none-to-minimal range. OSA subjects reported on average higher normal daytime sleepiness whereas controls were in the lower normal daytime sleepiness range. In contrast, although OSA subjects displayed significantly greater anxiety levels than controls, both groups had scores in the no or minimal anxiety range.

Dividing groups by sex revealed that females with OSA showed significantly higher levels of depressive symptoms and excessive daytime sleepiness, but not anxiety symptoms compared with female controls. In contrast, males with OSA showed higher levels of all symptoms compared with male controls. The distribution of ESS showed that the lower quarter for OSA was higher than the median for control (Fig. 1). In contrast, the distributions of GAD-7 and PHQ-9 showed that the lower quartiles for OSA and control were close to 0. The distribution of PSS showed that males only had a lower quartile for OSA that was higher than the control median.

**Figure 2:**
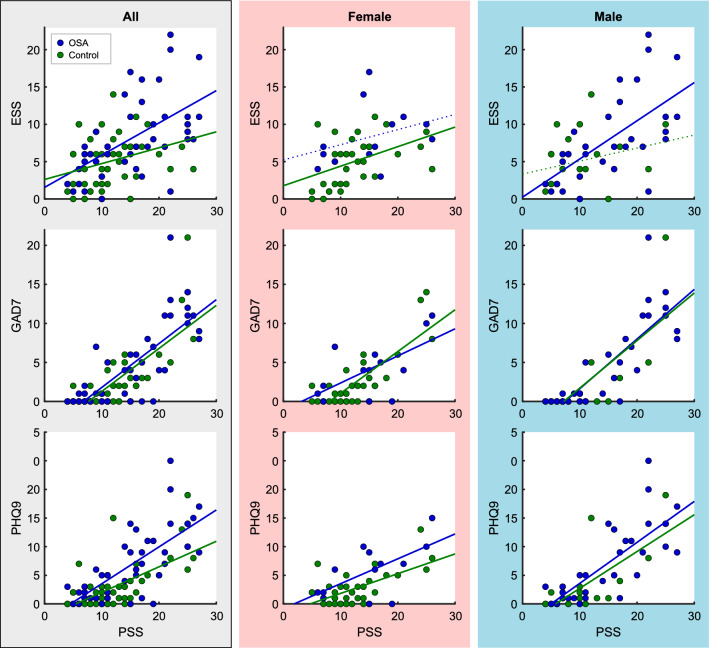
Symptoms vs. stress in OSA. Scatterplots of excessive daytime sleepiness (ESS), anxiety symptoms (GAD-7), and depressive symptoms (PHQ-9) versus stress (PSS) for combined sexes (All; left gray column), females (middle red column) and males (right blue column). Control values are green and OSA blue. Trend lines (with non-zero intercept) are plotted, with significant relationships indicated by solid (*P* ≤ 0.05) vs dashed (*P* > 0.05) lines. PSS = Perceived Stress Scale; GAD-7 = Generalized Anxiety Disorder scale; PHQ-9 = Patient Health Questionnaire; ESS = Epworth Sleepiness Scale.

Significant correlations occurred between the levels of stress (PSS) and ESS, PHQ-9 and GAD-7 measures in most groups (Table 2 and Fig. 2). The exceptions were in male controls, where the weaker correlation (R = 0.27) between PSS and ESS was not significant, and in female controls, where correlation (R = 0.23) between GAD-7 and ESS was not significant. The most robust correlations occurred between GAD-7 and PHQ-9 (R = 0.69 to 0.89). In all groups, there were strong correlations between PSS and GAD-7, and slightly lower levels between PSS with PHQ-9. ESS showed generally weaker correlations with other symptoms, and no significant correlations in female OSA. There were no significant differences in correlations between OSA and control groups.

**Table 2:** Correlation table for symptoms.

All	Control			OSA			P (OSA vs. Control)			
†	ESS	GAD-7	PHQ-9	ESS	GAD-7	PHQ-9	ESS	GAD-7	PHQ-9	
GAD-7	* .324			* .476			0.58			GAD-7
PHQ-9	* .467	* .794		* .531	* .890		0.92	0.88		PHQ-9
PSS	* .356	* .778	* .640	* .563	* .768	* .729	0.12	0.59	0.72	PSS

Female	Control			OSA			P			
	ESS	GAD-7	PHQ-9	ESS	GAD-7	PHQ-9	ESS	GAD-7	PHQ-9	
GAD-7	.228			.088			0.69			GAD-7
PHQ-9	* .388	* .689		.399	* .869		0.97	0.18		PHQ-9
PSS	* .444	* .814	* .623	.335	* .628	* .626	0.72	0.27	0.99	PSS

Male	Control			OSA			P			
	ESS	GAD-7	PHQ-9	ESS	GAD-7	PHQ-9	ESS	GAD-7	PHQ-9	
GAD-7	* .427			* .551			0.58			GAD-7
PHQ-9	* .544	* .884		* .565	* .894		0.92	0.88		PHQ-9
PSS	.270	* .753	* .720	* .627	* .814	* .768	0.12	0.59	0.72	PSS

OSA and control group Pearson’s correlation values are shown for OSA and control groups separately for combined-sex (All), female and male. * = significant correlation (*P* ≤ 0.05). Between-group P-values based on z-score comparisons of correlation coefficients.

^†^ *ESS* Epworth Sleepiness Scale, *GAD-7* Generalized Anxiety Disorder scale, *PHQ9* Patient Health Questionnaire, *PSS* Perceived Stress Scale. ♀ = female, ♂ = male.

Correlations of symptoms with sleep study parameters are shown in Table 3. No symptoms correlated significantly with REI or min SaO_2_, with the exception of a negative correlation of REI with PHQ-9 in females. Mean SaO_2_ showed moderate correlations (R = 0.43 to 0.48) with GAD-7, PHQ-9 and ESS in the combined and male groups, but not females. PSS was not correlated with any sleep study parameter.

**Table 3:** Correlation of sleep study parameters with symptoms in OSA.

†	All			Female			Male			
	REI	SaO_2_avg	SaO_2_min	REI	SaO_2_avg	SaO_2_min	REI	SaO_2_avg	SaO_2_min	
GAD-7	.17	*−.41	**−**.11	**−**.53	.02	.11	.28	*−.48	**−**.15	GAD7
PHQ-9	.15	*−.38	**−**.11	*−.60	**−**.06	.26	.27	*−.43	**−**.18	PHQ9
ESS	.03	*−.34	**−**.27	**−**.26	**−**.07	**−**.12	.09	*−.39	**−**.31	ESS
PSS	.09	**−**.22	**−**.05	**−**.44	.30	.26	.23	**−**.35	**−**.14	PSS

OSA group Pearson’s correlation values are shown for OSA and control groups separately for combined-sex (All), female and male. * = significant correlation (*P* ≤ 0.05) Not all control participants had a sleep study so these correlations were not calculated for those groups.

^†^*ESS* Epworth Sleepiness Scale, *GAD-7* Generalized Anxiety Disorder scale, *PHQ-9* Patient Health Questionnaire, *PSS* Perceived Stress Scale, *REI* respiratory event index, SaO_2_avg/min oxygen saturation average/minimum.

Raw data are available in a repository^31^.

## Discussion

Sleep apnea patients showed high levels psychological stress in addition to anxiety and depressive symptoms and excessive daytime sleepiness. Mean stress levels in OSA were in the moderate range. Stress symptoms were closely related to anxiety and depressive symptoms in all groups, and to a lesser degree excessive daytime sleepiness. Consistent with previous studies of psychological symptoms^1^, stress was associated with the presence but not severity of OSA. The combined findings demonstrate that psychological stress is a symptom present in OSA, and it is closely related to depressive and anxiety symptoms.

The higher levels of perceived stress in patients with OSA were expected, given the known links between stress and other OSA-related symptoms like anxiety and depression in non-OSA populations^32,33^. Consistent with other studies, we found high levels of anxiety and depression symptoms, as well as excessive sleepiness, in the OSA versus controls^2,3,34,35^. The neural circuits controlling stress and anxiety are closely related^36,37^, and we have previously shown damage in such circuits^21,22,38^. We showed specifically that both anxiety and depressive symptoms in OSA are related to neuroimaging markers of brain injury particularly in higher-order association areas such as the anterior cingulate, insular and prefrontal cortices^39,40^. There are numerous studies reporting anatomical changes in these same cortical regions in individuals with depressive and anxiety disorders^41,42^. Furthermore, chronic stress conditions are also associated with altered structure in the prefrontal and cingulate cortices^43–45^. Given this, it is likely that changes within these brain regions at least contribute to the altered psychological measures in OSA subjects reported here. One source of damage in OSA is likely intermittent hypoxia, and thus the relationship between mean SaO_2_ and stress may indirectly reflect greater brain injury leading to worsening symptoms^46,47^. However, it is not clear whether a lower resting SaO_2_ is related to hypoxic exposure during apneas. Furthermore, given the difference many days between recording of symptoms and the sleep study, and the inherent variability in apneic indices, the lack of observed correlation of stress with OSA severity indices should not be interpreted as showing such association does not exist.

Sex-specific patterns of some OSA symptoms have been noted previously, albeit in few studies^1^. Women with OSA have shown greater sleepiness than males with OSA, measured by ESS, which was consistent with another finding that demonstrated women with OSA are more impaired in daytime functioning and have greater sleepiness^1,48^. Possible factors can include genetic, hormonal, behavioral, and environmental factors. Additionally, sex differences in symptoms may related to health behaviors, such as likelihood to take preventative measures, use of prescription drugs, and acceptance of therapies^49^. However, the small number of female OSA participants limits the generalizability of the findings.

Earlier work showed higher levels of depressive symptoms in females than males, consistent with the general population^1,50^. However, our sample here showed that female patients with OSA did not report higher levels of depressive symptoms than OSA males. A previous study found that women without OSA showed more depressive symptoms than men, but that this difference was not present in OSA^50^. One possible reason for the difference in samples may be improvements in current procedures that exist for screening females for OSA as compared to even five years ago. Historically, OSA was seen as a male disease and females were less likely to be screened for the disorder, resulting in underdiagnosis of females relative to males^51^. Those recruited for the 2010 study were recruited from the UCLA Sleep Disorders Center in 2005. We also observed higher anxiety in OSA consistent with other studies^34^. However, the average GAD-7 was in the normal range (no or minimal anxiety) for this OSA and control sample. Our control females showed similar to lower anxiety symptoms than males, whereas the literature often shows it more typical for females to have higher anxiety levels and for men to show lower psychological symptoms^52–54^. Thus, anxiety symptoms are likely similar to depressive symptoms where the sample may not be representative of the general OSA population.

The relationship between stress, anxiety and depressive symptoms were present in both control and OSA groups. These relationships largely remained even when groups were divided by sex. It is possible that these significant relationships may not hold for mental health disorders. The above normal level of depressive symptoms reported here are not so high they must reflect a depressive disorder, and similarly high anxiety symptoms are not the same as an anxiety disorder. A diagnosable mental health disorder can reflect a more extreme and sustained negative psychological experience, and in some cases there are biological or traumatic underpinnings of such conditions.

One potential limitation is that the sample may have been healthier than the general OSA population, since exclusion criteria included common OSA comorbidities and risk factors, including weight > 125 kg, and current use of psychotropic medications. Such characteristics could have affected the results by biasing towards lower symptom scores, since comorbidities could create additional stress, and many people taking psychotropic medications would have high levels of stress-related symptoms. These trends would likely lessen the difference between OSA and control groups, and increase the chance of false negatives. Another limitation is that the sleep indices based on HSAT are inherently variable, and do not reflect the sleep status on the day of the psychological measures, so the lack of correlation of PSS with REI or min SaO_2_ may not generalize. Home sleep studies have a risk of underestimating sleep apnea severity, and hence may have missed mild OSA in the control group or led to lower indices in the OSA group^55^. Finally, the mean age of the OSA group was higher than the control group, and the sex-specific assessments were based on small sample sizes, so these findings would benefit from replication in other populations. The strength of the present study was the collection of multiple measures in the same participants, and it may be helpful when testing for OSA to assess stress in addition to the commonly-measured excessive daytime sleepiness and depressive symptoms.

In conclusion, we showed stress is elevated in OSA, similarly to sleepiness and depressive and anxiety symptoms. Contrasting other studies, females and males with OSA showed similar levels of all symptoms. In most groups, stress was strongly associated with depression and anxiety, but less so sleepiness. Our finding that stress is another symptom common to OSA raises the possibility of different treatment options than those focused on treating depressive or anxiety symptoms. For example, recommendations on reducing stress often include exercise, relaxing, recreation, socializing or participating in hobbies. Treatments for depression may focus on more medical interventions such as cognitive, behavioral, or psychotherapies, and commonly medications. Similarly, anxiety is often treated with medication. Since these three symptoms are closely aligned, treating stress in OSA may improve overall mental health.

## Data Availability

The data that support the findings of this study are openly available in Harvard Dataverse at http://doi.org/doi:10.7910/DVN/RWM03Y.
